# The Role of Informatics in Advancing Emergency Medicine: A Comprehensive Review

**DOI:** 10.7759/cureus.63979

**Published:** 2024-07-06

**Authors:** Anas Alhur

**Affiliations:** 1 Health Informatics, University of Hail College of Public Health and Health Informatics, Hail, SAU

**Keywords:** information technology, patient monitoring systems, interoperability, data analytics, telemedicine, clinical decision support systems, electronic health records, emergency medicine informatics

## Abstract

Emergency Medicine Informatics (EMI) is a rapidly advancing field that utilizes information technology to enhance the delivery of emergency medical services. This comprehensive literature review explores the key components, benefits, challenges, and future directions of EMI. By integrating Electronic Health Records, Clinical Decision Support Systems, telemedicine, data analytics, interoperability, and patient monitoring systems, EMI has the potential to significantly improve patient outcomes and operational efficiency in emergency departments. However, the implementation of these technologies faces several obstacles, including interoperability issues, data security concerns, usability challenges, and high costs. This review highlights how these technologies are transforming emergency care, discusses the barriers to their implementation, and provides perspectives on potential solutions and future progress in the field.

## Introduction and background

Emergency Medicine Informatics (EMI) is a rapidly evolving interdisciplinary field that leverages advanced information technology to enhance the efficiency and effectiveness of emergency medical services [1]. EMI encompasses a wide range of technologies, including Electronic Health Records (EHRs), Clinical Decision Support Systems (CDSS), telemedicine, data analytics, interoperability, and patient monitoring systems, all aimed at improving patient care, streamlining workflows, and enhancing clinical outcomes in emergency departments (EDs) [2].

The concept of EMI has transformed significantly over the past few decades. Initially, the focus centered on the basic digitization of medical records and the implementation of simple information systems to manage patient data [3]. However, innovations in technology have advanced the field into a more sophisticated era where complex data analytics, artificial intelligence (AI), and machine learning (ML) are utilized to support clinical decision-making, improve patient monitoring, and optimize resource allocation [4].

The adoption of EHRs in EDs has fundamentally altered the way patient information is stored, accessed, and shared [5]. EHR systems provide a comprehensive, real-time view of patient history, medications, allergies, and other critical data, thereby enhancing the accuracy of diagnoses and treatments. Studies have demonstrated that EHRs significantly reduce documentation errors and improve patient safety by ensuring that vital information is readily available to healthcare providers [6].

CDSS are designed to assist healthcare providers by offering evidence-based recommendations at the point of care [7]. These systems analyze patient data and provide alerts, reminders, and clinical guidelines to support decision-making processes. In emergency settings, CDSS can help reduce diagnostic errors, ensure adherence to best practices, and improve patient outcomes [8]. Research indicates that CDSS use leads to better compliance with clinical guidelines and improved patient care quality [9].

Telemedicine involves the use of telecommunications technology to provide medical care remotely. In emergency medicine, telemedicine enables swift consultation with specialists, remote monitoring of patients, and the provision of urgent care in rural or underserved areas [10]. The use of telemedicine has been shown to improve outcomes in critical situations, such as stroke and cardiac emergencies, by providing timely access to expert care [11].

Data analytics plays a crucial role in EMI by converting vast amounts of health data into actionable insights [12]. Predictive analytics uses historical data to forecast patient influx, disease outbreaks, and resource needs, allowing EDs to prepare and allocate resources effectively. Prescriptive analytics goes a step further by recommending specific actions to optimize clinical and operational performance [13]. The integration of big data and ML in emergency medicine is helping to identify patterns and trends that can lead to improved patient care and operational efficiency [14].

Interoperability refers to the ability of different information systems, devices, and applications to exchange and interpret shared data cohesively. Achieving interoperability in EMI is crucial for coordinated care, as it ensures that patient information flows seamlessly across various healthcare settings [15]. This enables clinicians to have a complete and accurate picture of a patient's health status, leading to better-informed clinical decisions. However, challenges such as varying standards, data formats, and privacy concerns need to be addressed to achieve true interoperability [16].

Advanced patient monitoring systems provide continuous, real-time data on patient vital signs, enabling clinicians to detect and respond to critical changes promptly [17]. These systems use sensors and wearable devices to monitor parameters such as heart rate, blood pressure, and oxygen saturation. In EDs, real-time monitoring is vital for managing critically ill patients and ensuring timely interventions. The integration of these systems with EHRs and CDSS enhances the overall quality of emergency care [18].

Importance and goals of EMI

The primary goal of EMI is to enhance the quality and efficiency of emergency medical care. By leveraging advanced information technology, EMI aims to improve patient outcomes, streamline operations, and enhance data-driven decision-making (Table 1).

**Table 1:** Importance and goals of EMI. EMI: Emergency Medicine Informatics, ED: Emergency department.

Importance and goals	Description	References
Improve patient outcomes	By providing clinicians with accurate, real-time information and decision support tools, EMI helps to reduce errors and improve the overall quality of care.	[1,2]
Streamline operations	Efficient data management and predictive analytics enable better resource allocation, reducing wait times and improving patient flow in EDs.	[4,19]
Enhance data-driven decision-making	Access to comprehensive data and advanced analytical tools allows healthcare providers to make more informed and effective clinical decisions.	[3,20,21]

This review aims to summarize the current state of EMI by examining its key components, benefits, challenges, and future directions. It highlights how EHRs, CDSS, telemedicine, data analytics, interoperability, and patient monitoring systems are transforming emergency care. Additionally, it discusses the barriers to implementing these technologies and provides perspectives on potential solutions and future advancements in the field.

## Review

Key components of *EMI*

Electronic Health Records (EHRs)

EHRs have become a cornerstone of modern healthcare, particularly in emergency departments (EDs) [22]. They provide a digital version of a patient's paper chart, offering real-time, patient-centered records that make information available instantly and securely to authorized users [23]. EHRs enhance the quality of care by improving the accuracy of patient records, facilitating the continuity of care, and reducing medical errors [24]. Studies have demonstrated that the use of EHRs in EDs leads to significant improvements in documentation accuracy and patient safety, particularly by reducing medication errors and ensuring that critical patient information is always available to healthcare providers [6,22,25,26].

Clinical Decision Support Systems (CDSS)

CDSS are designed to aid clinical decision-making by providing evidence-based knowledge in the context of patient data. These systems offer various tools, such as alerts, reminders, clinical guidelines, diagnostic support, and treatment suggestions, to assist healthcare providers in making timely and informed decisions [7]. In emergency settings, where time is critical, CDSS can help reduce diagnostic errors, improve adherence to clinical protocols, and enhance patient outcomes. Research indicates that CDSS use leads to better compliance with clinical guidelines and overall improvements in patient care quality. For example, systematic reviews have highlighted the effectiveness of CDSS in improving clinical processes and patient outcomes in emergency departments [9].

Telemedicine

Telemedicine involves the use of telecommunication technology to provide healthcare services remotely. It has proven particularly valuable in emergency medicine by enabling remote consultations, diagnostics, and treatment, especially in rural or underserved areas [10]. Telemedicine can facilitate timely access to specialist care, which is crucial in emergency situations such as strokes, cardiac events, and trauma cases [27]. The implementation of telemedicine has been shown to improve patient outcomes by reducing the time to treatment and enabling continuous monitoring and follow-up care [28]. Research has demonstrated the positive impacts of telemedicine on emergency care, particularly in improving access to specialist consultations and enhancing patient management in remote areas [11].

Data Analytics

Data analytics in emergency medicine informatics involves analyzing large datasets to extract actionable insights. Predictive analytics uses historical data to forecast future events, such as patient admission rates, disease outbreaks, and resource needs, allowing EDs to prepare and allocate resources more effectively [29,30]. Prescriptive analytics goes further by recommending specific actions to optimize clinical and operational performance. The use of big data and ML in emergency medicine is helping to identify patterns and trends, leading to improved patient care and operational efficiency [12]. For example, predictive analytics models have been used to forecast in-hospital mortality rates for sepsis patients and optimize resource allocation in emergency departments [13,31].

Interoperability

Interoperability is the ability of different health information systems and software applications to communicate, exchange, and interpret shared data effectively. Achieving interoperability in emergency medicine informatics is crucial for coordinated patient care, as it ensures seamless data exchange between various healthcare providers and systems. Standards like HL7 FHIR (Fast Healthcare Interoperability Resources) are being adopted to facilitate seamless data exchange. However, challenges remain, such as varying implementation practices and data formats. For instance, while HL7 FHIR is widely accepted, some institutions face difficulties in fully integrating it due to legacy systems and inconsistent data standards across different platforms [15,16].

Patient Monitoring Systems

Advanced patient monitoring systems use sensors and wearable devices to continuously monitor vital signs and other critical parameters in real-time. These systems provide clinicians with timely alerts about changes in a patient's condition, enabling prompt intervention [17]. In EDs, real-time monitoring is essential for managing critically ill patients and ensuring timely responses to life-threatening conditions. The integration of these systems with EHRs and CDSS enhances the overall quality of emergency care by providing comprehensive and up-to-date patient information. Studies have shown the feasibility and effectiveness of advanced monitoring systems in improving patient management and outcomes in emergency settings [18,32].

Benefits of EMI

The integration of informatics into emergency medicine offers numerous benefits.

Enhanced Patient Safety

By reducing errors and improving the accuracy of diagnoses and treatments, EMI enhances patient safety. For instance, a study conducted found that the implementation of EHRs reduced medication errors by 30% within the first year [2].

Improved Efficiency

Informatics solutions streamline workflows and reduce the time to diagnosis and treatment. For example, in 2020, findings demonstrated that predictive analytics reduced patient wait times by 20% by optimizing resource allocation and improving patient flow [21].

Better Resource Management

Advanced data analytics and real-time monitoring systems allow for better management of ED resources, such as staff, equipment, and beds, ensuring they are used optimally to meet patient needs. Informatics tools facilitate the efficient allocation of these resources, enhancing overall operational efficiency [19].

Data-Driven Decision-Making

Access to comprehensive data and advanced analytical tools enables healthcare providers to make more informed and effective clinical decisions, improving the overall quality of care. The implementation of AI and ML in EDs aids in better decision-making processes. For example, researchers found that ML algorithms improved the accuracy of triage decisions, resulting in a 10% reduction in unnecessary admissions [13,33].

Challenges in EMI

Despite its numerous benefits, the implementation of EMI faces several challenges.

Interoperability Issues

Achieving seamless data exchange between disparate systems remains a significant challenge. Variations in data standards and formats can hinder effective communication and coordination among different healthcare providers. Addressing these interoperability issues requires a concerted effort to standardize data formats and protocols [4].

Data Security and Privacy

Protecting patient data from breaches while maintaining accessibility is a critical concern. Ensuring robust data security measures and compliance with privacy regulations is essential to gain and maintain patient trust. The implementation of secure and compliant informatics systems is crucial for protecting sensitive patient information [34].

Usability Designing

Systems that are user-friendly and intuitive for clinicians working in high-pressure environments are crucial. Complex or poorly designed interfaces can lead to user frustration and decreased efficiency. Effective usability testing and end-user feedback are essential for successfully implementing these systems [35-37].

Implementation Costs

The high costs associated with deploying and maintaining advanced informatics systems can be a barrier, particularly for smaller or resource-constrained healthcare facilities. Securing funding and demonstrating the return on investment is critical for successful implementation. Addressing cost concerns involves strategic planning and investment in scalable solutions [38].

Future directions

Future research and development in EMI should focus on addressing the current challenges and exploring new opportunities for improvement. Key areas for future advancements include enhancing interoperability, advanced analytics, telemedicine expansion, user-centered design, and data security (Table 2).

**Table 2:** Future directions in EMI. EMI: Emergency Medicine Informatics.

Key area	Description	References
Enhancing interoperability	Developing universal standards and protocols for data exchange to facilitate seamless communication between different health information systems.	[15,16]
Advanced analytics	Leveraging artificial intelligence and machine learning to enhance predictive capabilities and support more sophisticated decision-making processes.	[2,12]
Telemedicine expansion	Broadening the scope and accessibility of telemedicine services to reach more patients, particularly in underserved areas.	[10]
User-centered design	Creating more intuitive and efficient interfaces for clinicians to improve usability and adoption rates.	[39]
Data security	Strengthening measures to protect patient information, including the use of advanced encryption techniques and robust cybersecurity frameworks.	[34,40]

## Conclusions

Emergency Medicine Informatics (EMI) integrates information technology with emergency medical services, promising improved patient outcomes and operational efficiency. Despite challenges like interoperability issues and high implementation costs, ongoing research and development in EMI can address these obstacles. By overcoming these hurdles, EMI has the potential to significantly enhance the quality and efficiency of emergency healthcare delivery.
